# Solar Energy Harvesting to Improve Capabilities of Wearable Devices

**DOI:** 10.3390/s22103950

**Published:** 2022-05-23

**Authors:** Alba Páez-Montoro, Mario García-Valderas, Emilio Olías-Ruíz, Celia López-Ongil

**Affiliations:** Department of Electronic Technology, Universidad Carlos III de Madrid, 28911 Madrid, Spain; mgvalder@ing.uc3m.es (M.G.-V.); olias@ing.uc3m.es (E.O.-R.); celia@ing.uc3m.es (C.L.-O.)

**Keywords:** energy harvesting, internet of things, physiological sensors, solar energy, wearables, wireless communication, wireless sensor network

## Abstract

The market of wearable devices has been growing over the past decades. Smart wearables are usually part of IoT (Internet of things) systems and include many functionalities such as physiological sensors, processing units and wireless communications, that are useful in fields like healthcare, activity tracking and sports, among others. The number of functions that wearables have are increasing all the time. This result in an increase in power consumption and more frequent recharges of the battery. A good option to solve this problem is using energy harvesting so that the energy available in the environment is used as a backup power source. In this paper, an energy harvesting system for solar energy with a flexible battery, a semi-flexible solar harvester module and a BLE (Bluetooth^®^ Low Energy) microprocessor module is presented as a proof-of-concept for the future integration of solar energy harvesting in a real wearable smart device. The designed device was tested under different circumstances to estimate the increase in battery lifetime during common daily routines. For this purpose, a procedure for testing energy harvesting solutions, based on solar energy, in wearable devices has been proposed. The main result obtained is that the device could permanently work if the solar cells received a significant amount of direct sunlight for 6 h every day. Moreover, in real-life scenarios, the device was able to generate a minimum and a maximum power of 27.8 mW and 159.1 mW, respectively. For the wearable system selected, Bindi, the dynamic tests emulating daily routines has provided increases in the state of charge from 19% (winter cloudy days, 4 solar cells) to 53% (spring sunny days, 2 solar cells).

## 1. Introduction

**Figure 1:**
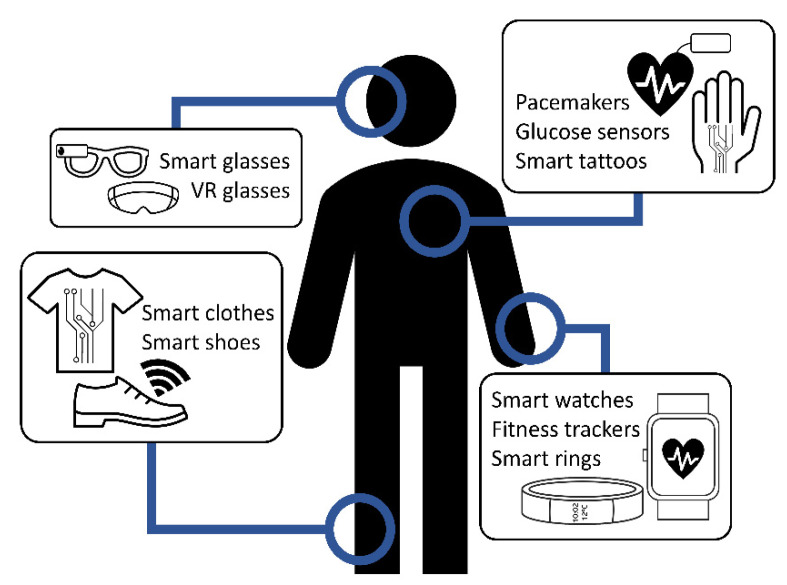
Examples of wearable devices.

Wearables are smart devices that are usually worn as watches, bands, clothes or even skin implants. In Figure 1, some examples are presented. These devices normally include one or more sensors, a power source, a processing unit and wireless communication modules. The most common applications of these smart devices are healthcare [1], activity tracking and sports [2], education [3], etc.

The wearable devices market has been growing over the past few years. According to [4], this market will reach 150 billion EUR by 2028. Recently, due to the COVID-19 outbreak, the use of wearable devices has also increased, since many of them include physiological sensors and are helpful for remote patient monitoring [5].

Wearables are usually part of the Internet of Things (IoT) systems. IoT provides a platform to connect and exchange information between multiple elements, from wearable sensors that collect information from the physical world to remote servers that store data and/or command different actions [6]. The combination of wearable devices with communication and computing capabilities results in a Cyber-Physical System (CPS) [7].

This paper is focused on testing the viability of including energy harvesting techniques in a smart bracelet, which is part of an IoT system called Bindi (presented in [8]). Bindi consists of a bracelet and a pendant that collect physiological and acoustic data and send them to a mobile phone application (app), which is connected to a remote server. Bindi has been made to protect women against gender-based violence by automatically detecting fear with the measurements from smart sensors and embedded machine learning algorithms.

**Figure 2:**
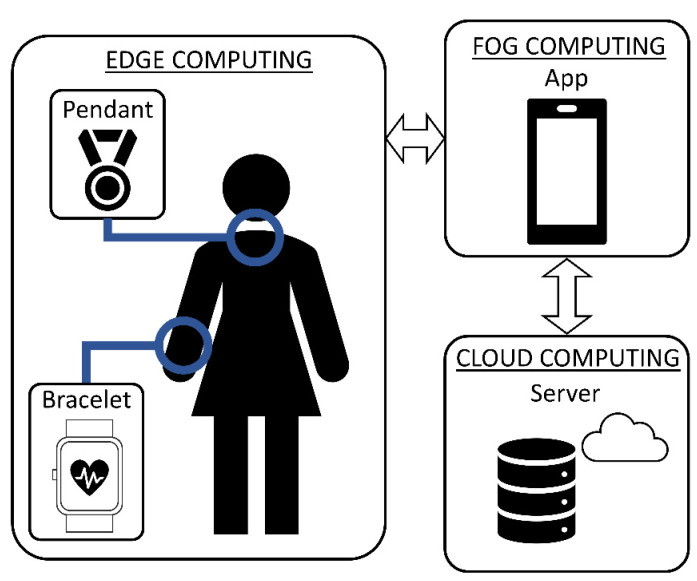
Bindi architecture as an IoT system.

According to [9], an IoT system can be divided into three main layers with different data computing capabilities: cloud, fog and edge. In Bindi, cloud computing is conceived as the remote server, fog computing as the mobile application and edge computing as the wearable devices. In Figure 2, the architecture of Bindi and the IoT layers for each component are shown.

**Figure 3:**
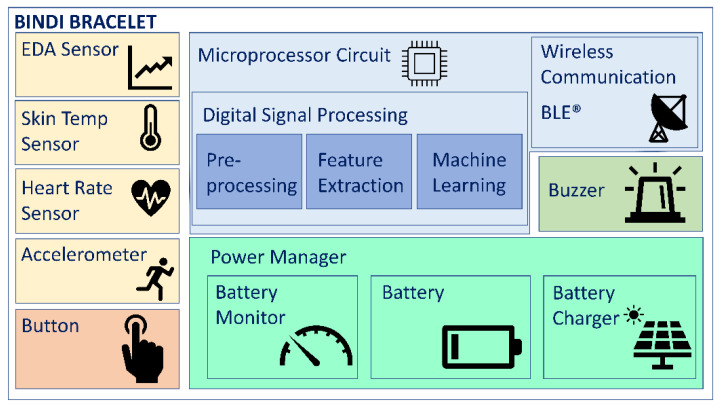
Bindi bracelet schematic.

The smart bracelet in Bindi has three physiological sensors: a heart rate sensor, an electrodermal activity sensor (EDA) and a temperature sensor. The sampling frequencies are 100 Hz, 10 Hz and 5 Hz, respectively. The data collected from them are processed (noise elimination filtering, artifacts removal, signal quality assessment, band frequencies selection, main feature extraction and machine learning algorithms), encrypted and sent wirelessly through a BLE (Bluetooth^®^ Low Energy) protocol with a data rate of around 1 kBps. Even though the bracelet can automatically detect fear by using the embedded machine learning algorithms, ciphered raw data from the sensors are sent so that the physiological signals can be kept in the server and can be used as judicial evidence in gender-based violent crimes. The bracelet also has other functionalities, such as a panic button, an accelerometer, a buzzer or power management, that can be seen in Figure 3.

The system includes a low power microprocessor, from Nordic Semiconductor^®^ (nRF52840), which manages the sampling schedule for sensors, the I2C and SPI intranode communications, the tasks related to BLE wireless protocol including pairing, command reception and processing, as well as data continuous streaming and battery monitoring. The system implies a firmware of 512 kB of RAM and 64 kB of Flash memory, including the BLE stack. The average power consumption of the bracelet during its normal operation, three physiological sensors, accelerometer, battery monitoring and wireless data streaming, is 18.5 mW (5 mA, 3.7 V).

So many capabilities have a direct impact on the power consumption of the bracelet, and, in consequence, its battery life. The longer the battery life, the better the user experience, since the wearable device will not need to be charged as frequently, making it easier to use and more comfortable.

Wearable devices are powered by electrochemical Energy Storage Devices (ESDs), such as supercapacitors or rechargeable batteries, since these are efficient and clean technologies. Nowadays, rechargeable batteries lead the wearable market since they have a higher energy density than supercapacitors. Moreover, supercapacitors are traditionally larger and it is hard to integrate them within a wearable device. Even though a lot of research has been done into new experimental, flexible and small-sized supercapacitors, they are still inadequate for large scale, efficient fabrication [10].

Ideally, it would be more convenient to have wearable devices that can be totally powered with the energy available in their surroundings. Energy harvesting technologies are used to collect energy from the environment and use it to power a device. The most common energy harvesting sources are solar energy, thermal energy and mechanical energy. However, it is not possible to power a device only by using energy harvesting with the currently available technologies. Energy harvesters such as solar cells (which obtain power from the sunlight) or Thermoelectric Generators (TEGs) (which obtain power from temperature differences) have very low efficiencies and the energy they harvest is not always available (e.g., solar cells in the night). Hence, they are used as backup sources that work alongside rechargeable batteries. Using two or more energy sources in a hybrid energy harvesting system is also a good option, since one power source can back up the others when they are not available [11].

Even though solar energy is not always available, it has become the most used energy source for energy harvesting applications in wearables. Photovoltaic technologies have been developing since the discovery of the photovoltaic effect in 1839 by Becquerel [12]. There are three generations of solar cells, from the first generation of silicon solar cells to the second generation of III-V composites, to the third generation of perovskite, dye-sensitized, quantum dot and organic solar cells [13].

In summary, there must be a trade-off between wearable device capabilities and their consequent power consumption. The objective of this work is to design a solar energy harvesting system and test the viability of including solar cells in the smart bracelet of the Bindi system, to cope with its many capabilities. Solar cell performance depends on many factors, such as the operating conditions of the load (sensor measurements and connectivity), time, location and position of the solar cells, and it is difficult to theoretically estimate the generated power when they are included in a wearable device. Thus, the objective of this work is also to test the performance of the solar energy harvesting system included in a wearable device by using different daily life scenarios. Commercial devices will be used in this design, since they are more mature technologies and most energy harvesting wearable devices in the literature are experimental and do not consider large-scale production and fabrication costs [14]. Technology accessibility in the mid-term is a condition for Bindi design.

## 2. Related Work

Using energy harvesting not only increases the battery life of portable devices with the energy available in the environment, but it also reduces the ecological footprint compared to fossil fuels, the main power source used worldwide [15,16].

Solar energy is the most common energy source used in energy harvesting systems since it is possible to obtain tens or even hundreds of mW with relatively small-sized solar cells [17].

Solar cells are made of semiconductor junctions that can convert sunlight energy into electrical energy via the photovoltaic effect. Semiconductors have a characteristic energy level called a band gap. When incident photons have higher energy than this band gap, the solar cell will generate an electrical current.

Solar cells have a theoretical efficiency limit (called the Shockley–Queisser limit) at 33.7% on an ideal semiconductor with a band gap of 1.34 eV. This limit was calculated considering that all photons with energies higher than the band gap of the semiconductor are absorbed, but not all of them can be converted to electrical energy, since the band gap can change due to the temperature increase. Furthermore, only radiative recombination was considered. In real solar cells, there are more factors that affect the efficiency, so the actual limit is lower than the theoretical Shockley–Queisser limit (around 29% [18]). To overcome this intrinsic limit, exhaustive research into new materials and configurations must be done.

Nowadays, more than 90% of solar cells available in the market are silicon solar cells, due to their higher efficiency levels and their relatively low production costs [18,19]. Silicon solar cells can be divided into three groups depending on their internal structure: monocrystalline, polycrystalline and amorphous. Monocrystalline silicon solar cells have the highest efficiency, with a reported value of 26.7% [18]. On the other hand, amorphous silicon is mainly used to manufacture flexible solar cells, which are a good option for wearable devices in terms of comfort. However, their efficiency levels are still very low (around 12%).

Other semiconductor materials, such as GaAs, can also be used to manufacture solar cells. In [20], Alta Devices Inc. demonstrated an efficiency of 27.6% in a single junction GaAs thin-film solar cell. However, this semiconductor needs a good quality encapsulation when used in wearable devices due to the toxicity of the arsenic. On the other hand, it is possible to use more than one semiconductor junction (heterojunction solar cells), such as the one presented by LONGi Inc. with an efficiency of 26.3% [21]. Organic solar cells, like the one presented by Epishine [22], are also being studied, since they have potential for both roll-to-roll fabrication and good performance with indoor lighting. Recently, perovskite solar cells have achieved an efficiency value similar to silicon solar cells and it is estimated that the production costs would be halved. However, the stability of perovskite solar cells is still a challenge, since they suffer from fast degradation when exposed to typical ambient conditions [23,24].

**Figure 4:**
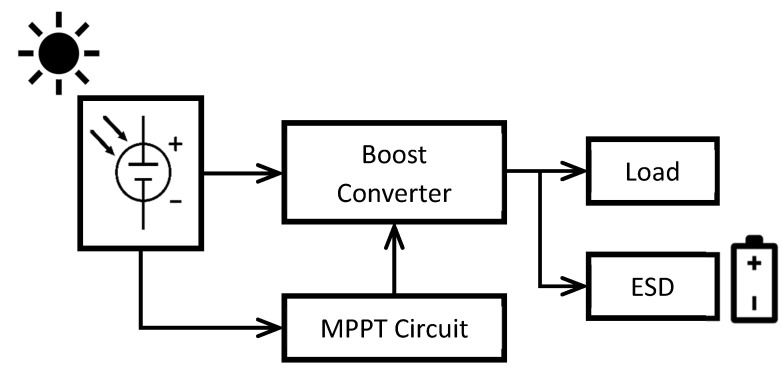
Schematic of a typical solar energy harvesting circuit.

Energy harvesting circuits usually follow the same structure. The input from the harvester goes to a conditioning circuit that optimizes the generated power, then the energy is stored in an ESD or is used to power an electronic load. Solar energy harvesting is no exception. The schematic shown in Figure 4 is a representation of the common structure for solar energy harvesting circuits. The input from the solar cell goes through a boost converter controlled by a Maximum Power Point Tracking (MPPT) algorithm. MPPT algorithms force the solar cells to work around their Maximum Power Point (MPP), which is normally at 80% of their open circuit voltage. The energy obtained from the solar cell is used to power a load or is stored in an ESD.

As previously stated, in wearables, the load is typically an electronic system that includes one or more sensors, a processing unit and wireless communication devices. The ESD is usually a rechargeable battery whose chemical composition depends on the application.

**Figure 5:**
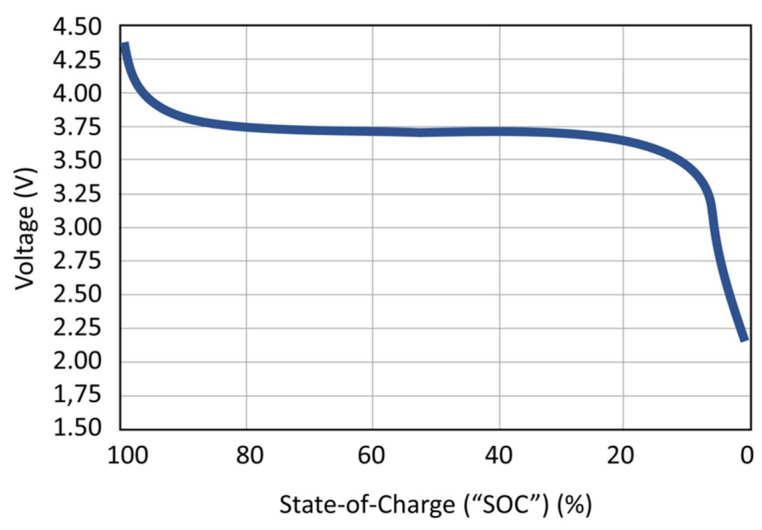
Typical Li-ion discharge curve [25].

In the 20th Century, NiCd rechargeable batteries were used to power portable devices, but cadmium is an expensive and toxic component, so they were mostly replaced by NiMH rechargeable batteries, which have a higher power density and a lower toxicity. Nowadays, most commercial wearable devices use Lithium-ion (Li-ion) or Lithium-ion polymer (LiPo) rechargeable batteries. Both of them have good values of power density and they can maintain a constant voltage (called nominal voltage) during almost all of the discharge, as seen in Figure 5 [25]. Moreover, it is possible to manufacture thin and flexible Li-ion and LiPo batteries. However, their main disadvantage is that they may explode when overcharged, so designers must be especially cautious and include battery protection circuits in their systems [26].

In this work, a solar energy harvesting system is designed to test the viability of including commercial monocrystalline silicon solar cells and a LiPo flexible battery in a wearable device with a high number of functionalities. Previous to this work, other energy harvesting applications in wearables have been studied from the literature so that the proposed system could be compared to other systems.

In [27] Toh et al. use a flexible solar cell with an MPPT circuit and a supercapacitor to power a wearable temperature sensor and a radio frequency transceiver. The wearable is able to work only with the energy harvested from the solar cells whenever it is not transmitting data from the temperature sensor. During the transmission, the average power consumption of the system is 3.91 µW and it needs to be powered from the supercapacitor. The device can work for 15 h since body temperature data are only sent once every minute.

In [28], Kim et al. use a TEG and an LiS battery to power a commercial glucose sensor with a power consumption of 64 µW. TEGs do not have high voltage levels when used in the human body due to the low temperature difference between the skin and the ambient. Thus, using an LiS battery is a good option, since its typical voltage is around 2 V, which is lower than the typical voltage in Li-ion and LiPo batteries (3.7 V). Moreover, LiS batteries have a higher energy density than Li-ion batteries. Nevertheless, they deteriorate faster, so they are not ready to replace commercial Li-ion batteries [29].

As for mechanical energy, in [30], Nastro et al. designed energy harvesters for wearables based on piezoelectric cantilevers. Piezoelectric materials generate power from mechanical energy, but they often need high frequency excitation. Since human movements are in the order of a few Hz, in this work, the authors use two and four piezoelectric cantilevers to improve the power generated by two different devices at low frequencies. They consist of an internal steel ball which moves when the user is moving and impacts on the piezoelectric cantilevers. They were able to generate a maximum instant power of 1.58 mW with an average power of 9.65 µW in 0.7 s when wearing the designed devices as a wristband and when moving the hand at a low frequency.

The average power values generated by the devices presented in [27,28,30] are in the order of µW, which is enough for wearables with only a few functionalities, such as the presented glucose sensor or the temperature sensor that sends one sample every minute. However, wearable smart devices include more functionalities, so these proposals would not be able to significantly impact on their power consumption.

On the other hand, a great amount of work has been done on the development of hybrid energy harvesting devices. The hybridization of two or more power sources can be done within the energy harvester itself or by using independent energy harvesters in one wearable device.

One hybrid system example is the jacket designed by Brogan et al. that includes 16 solar cells and 12 TEGs to charge a battery pack [31]. They obtained a maximum power output of 500 mW in a sunny day and outdoors. Note that the power generated from the solar cell was the main contribution to the total power, since power from the TEGs was on the order of the microwatts µW.

In [32], Magno et al. present a low power smart bracelet that combines various sensors with solar and thermal energy harvesting. Both the solar cells and TEGs are embedded in the band of the bracelet. They can generate up to 550 µW, with the solar cells indoor and up to 250 µW with the TEGs and a difference of 5 K.

In [33], Lemey et al. present a hybrid textile module that consists of a textile antenna integrated with a TEG and two solar cells to harvest solar energy in indoor and outdoor scenarios. When a user moves into a room with artificial light at 17.5 ºC, the harvesters can generate up to 310.9 µW with a total system efficiency of 47.7%. Using the device in an outdoor scenario increases considerably the generated power up to 46 mW.

Another example is [34], where Liu et al. present a photo-thermo-electrochemical device. This device consists of a TEG covered with a photothermal polymer that improves solar light absorption and, thus, creates a higher temperature difference for the TEG to generate power. They can charge a supercapacitor over 250 mV. Even though the device has a good performance and the production costs were considered, wearable smart devices normally use rechargeable batteries, which work at a higher voltage than hundreds of mV.

Moreover, in [35], Ren et al. present a hybrid harvester for solar and mechanical energy. The device includes both a flexible organic solar cell and a triboelectric generator, which can generate power from the contact of two materials. The harvester is covered with a layer of groove-shaped micro/nanostructured haze thin film, which is a hydrophobic material that improves light trapping, triboelectric effect and encapsulation.

Another hybrid harvester is designed in [13] using organic solar cells and TEGs. Organic solar cells are usually not considered for commercial products, since their efficiency is lower than that of other solar cell technologies. However, the authors of this work increased the collected electrical energy by 46.3% for a temperature difference of 10 K.

After the analysis of the state-of-the-art energy harvesting systems, it was decided that commercial solar cells and a commercial flexible battery would be used. Even if some of the examples previously presented have good results, they are mainly experimental devices, meaning that production costs and long-term stability have not been considered. Furthermore, the energy harvesting systems presented in these studies are mainly integrated with low-power wearables that do not have a high number of functionalities, which is not the case in the study selected for this work, Bindi.

## 3. System Design

In this work, an electronic system has been designed as a proof-of-concept to test and validate a solar energy harvesting application proposal. Specifically, it has been made so that a microprocessor module with a power consumption similar to a smart wearable could be powered by a flexible LiPo battery and solar cells. Moreover, the device also offers the possibility of recharging the battery through a USB (Universal Serial Bus) charger in case a faster charge is needed. This system is intended to be included in a Bindi smart bracelet.

**Figure 6:**
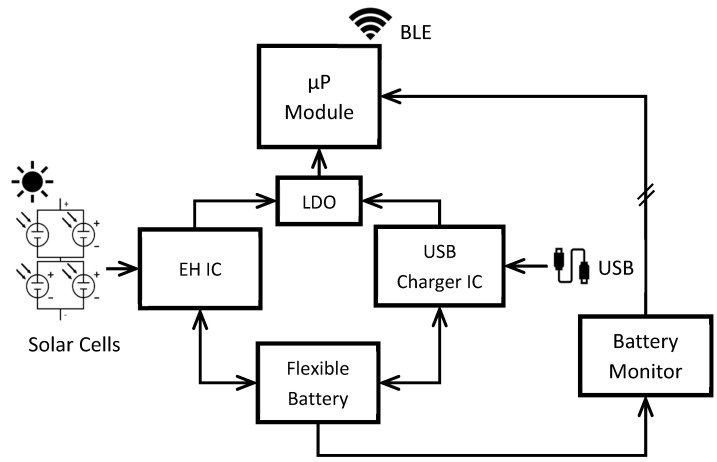
Schematic of the designed device.

The schematic with the basic electronic components of the system is presented in Figure 6. They are described throughout the rest of this section.

The microprocessor module (µP Module in Figure 6) is the Aconno^TM^ ACN52840, which is especially made for IoT applications. It includes the Nordic Semiconductor^®^ nRF52840 SoC (System on Chip) and an on-board antenna for BLE communication, alongside other features. The SoC is the same as the one used in Bindi wearables. The module can be powered by a voltage between 1.7 V and 3.6 V. More information relating to the module can be found in [36].

Two power management Integrated Circuits (ICs) made by Texas Instruments^®^ (Dallas, TX, USA) are used to recharge the battery and to power the microprocessor module. In Figure 6 they are presented as EH IC (for the Energy Harvesting power supply) and USB Charger IC (for the USB charging). They are never enabled at the same time, so a manual switch is used to select between them. Furthermore, both ICs include safety measures for battery charging such as overvoltage protection or charging current control.

The EH IC (BQ25570) is used to manage the power acquired from the solar cells to both charge the battery and power the microprocessor module. This IC includes an MPPT algorithm that can be configured depending on the energy source that is being used. It can normally charge an ESD with an input power as low as 5 µW connected to its boost converter. Moreover, the IC can power a load at a fixed output voltage value with its buck converter. The output voltage (VOUT) and other parameters such as a battery good indicator (VBAT_OK) and overvoltage threshold (VBAT_OV) can be configured with a resistor divider. In this case, VOUT is set to 1.7996 V, the VBAT_OK threshold was set to 2.823 V and 3.097 V (two different values due to charging/discharging hysteresis) and VBAT_OV was set to 4.308 V. If the power from the solar cells is high enough, the load is powered and the battery is recharged by them. If the power is too low, the load is powered directly by the battery. More information about this IC can be found in [37].

The USB Charger IC (BQ24232) is used to recharge the battery and power the load when an external Direct Current (DC) source is connected through a type-C USB connector. The output voltage when a DC source is connected is 4.4 V and cannot be changed. The maximum charging current is programmed with a resistor and it is set to 136 mA. In case there is no DC external source connected, the load is powered by the battery. More information about this IC can be found in [38].

In this design, the load (µP Module) is powered by 1.8 V. Since the USB Charger IC has an output voltage of 4.4 V, voltage regulation is needed. A Low-Dropout Voltage regulator (LDO) was used. The selected LDO is the same used in Bindi system: the Microchip Technology^®^ MIC5365 has a fixed output voltage of 1.8 V. Although the EH IC does not need voltage regulation (since the output voltage is 1.7996 V), it was decided that its output would be also connected to the LDO input so that manually switching between the two ICs is easier. More information can be found in [39].

As for the battery, a Grepow^®^ flexible LiPo battery with a capacity of 135 mAh and a nominal voltage of 3.7 V is used [40]. In addition to the USB Charger IC and the EH IC, the battery also includes a protection circuit to prevent overcharging. Even though LiPo batteries can be dangerous, they are leading the wearable market. Safer batteries are mostly experimental prototypes, which are not ready to be manufactured on a large scale. For these reasons, it was decided to use a LiPo battery in the proposed system, taking care of the overcharging protection and considering a good encapsulation design as future work. Furthermore, the selected battery is flexible, so it is a good choice for a wearable device.

A battery monitor is also needed to measure the battery current and the State-of-Charge (SOC). The Maxim Integrated^TM^ Fuel Gauge IC MAX17055 [41] has been selected since it is also used in the wearable devices in Bindi. This IC uses the Maxim ModelGauge^TM^ m5 EZ algorithm for battery measurements with tolerance against lithium batteries diversity. It uses an I2C interface to communicate with the microprocessor.

The selection of the solar cells used for this device is based on theoretical calculations. These calculations have been made considering the following:

- In the place where the experiments are made (Madrid, Spain), there are 8 h of light and 16 h of no light during a day, on average.
- During the 8 h of light time, the solar cells would fully power the device while recharging the battery for the remaining 16 h of no light.
- During the 16 h of no light, the device would be fully powered only by the battery.
- The average current drawn by the device is 5 mA, which is similar to the power consumption of the smart bracelet in Bindi, according to their battery monitor, when the bracelet is in continuous operation: receiving and processing data from sensors and wireless data streaming.
- The voltage provided by the battery is 3.7 V (nominal voltage).
- The typical efficiency of a solar cell is 25%.

Taking these into consideration, the energy required to power the device during the day and recharge the battery for 16 h of no light is the power of the device multiplied by the 24 h in a day. Considering that the power can be obtained as the supply voltage of a system multiplied by the drawn current:

(1) $$E=V_{nom}\cdot I_{avg}\cdot t_{day},$$

where *E* is the energy required by the device during a day, *V_nom_* is the nominal value of the battery voltage, *I_avg_* is the average current drawn by the device and *t_day_* is the 24 h in a day.

Hence, the energy required would be 1598.4 J. However, this value of energy should be acquired by the solar cell during the 8 h of light, meaning that their power (energy divided by time) should be:

(2) $$P_{sc}=\frac{E}{t_{light}\cdot 3600\cdot \eta },$$

where *P_sc_* is the power given by the solar cell, *E* is the energy required by the device during a day, *t_light_* is the hours of light during a day and η is the solar cell efficiency.

Therefore, the solar cell should be able to supply 222 mW. After obtaining this value, the ANYSOLAR Ltd. monocrystalline solar cells SM141K04LV were selected [42]. They have a maximum power of 123 mW and an efficiency of 25%. Four of these solar cells were connected in a series-parallel configuration, so that the total power is four times higher than the power of one solar cell. The configuration can be seen in the schematic of the device in Figure 6. Even though they are rigid solar cells, they are connected by soldered wire, so the result is a 60 × 45 mm for a semi-flexible module.

**Figure 7:**
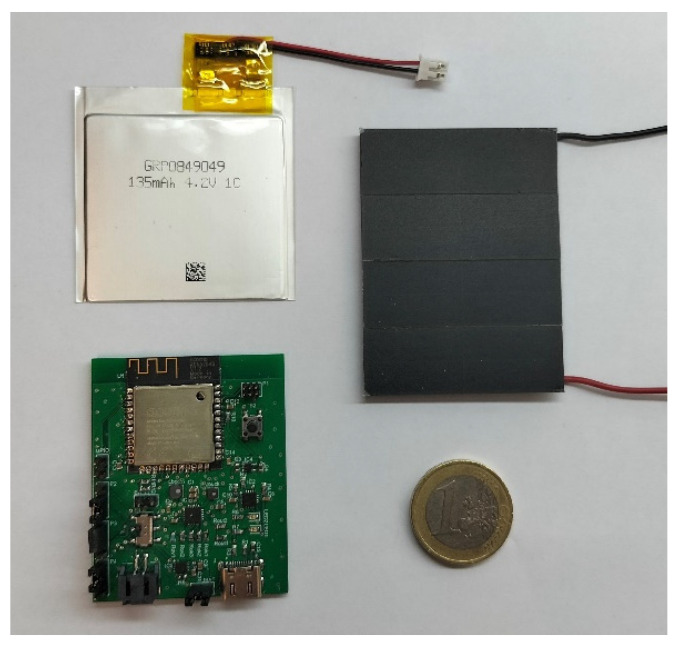
Parts of the designed device. Flexible battery (upper left), PCB (lower left) and solar cells module (right). 1 EUR coin used as a size reference.

Using amorphous silicon flexible solar cells was also considered but, since their efficiency is lower than monocrystalline silicon solar cells, this consideration was discarded. The entire circuit (except for the battery and the solar cells) was integrated in a PCB (Printed Circuit Board). The PCB, the flexible battery and the solar cells module that form the complete device are presented in Figure 7. The dimensions of these components are 48 × 48 mm^2^, 60 × 45 mm^2^, 43 × 53 mm^2^ for battery, solar cells module and PCB board, respectively.

## 4. Testing Procedure

The testing procedure for wearable devices using energy harvesting from the solar panels considers the different conditions of sunlight and user’s activity, which is related to the periods of solar exposure and wireless protocol connectivity. Two different types of test have been defined. The first set of tests was devoted to measuring the static conditions for the device, except for the sunlight received. The experiments consisted of placing the solar cells in specific positions for the entire discharge time to observe their performance and contribution to the battery life in different sunlight conditions. These tests were aimed mainly at measuring the capacity of the solar panels to recharge the battery under different sunlight conditions. The second set of tests was aimed at measuring the behaviour of the energy harvesting module under dynamic conditions. The whole system was worn by the user during her daily routines, while the state of charge of the battery was sent wirelessly to a smart phone. A 3D-printed flexible module has been prototyped to contain the designed PCB and the flexible battery to allow easiness and comfort of use.

It is difficult to state the standard dynamic conditions for testing the performance of an energy harvesting proposal. In this work, the authors have proposed a set of typical daily routines attending to the application of the wearable system selected, Bindi. The procedure for assessing the energy harvesting capacity may differ for another application, but the two types of tests proposed should be performed in different conditions.

In both tests, the processing unit of the designed device is loaded with a firmware that allows the battery monitor to measure the battery current in mA (milliamps) and SOC in % and send them to a mobile phone through a BLE wireless protocol every second.

### 4.1. First Set

In the first set of tests, the solar cells were manually laid so that the light would hit in different ways. A lux meter 5032C from MAVOLUX^®^ was placed next to the solar cells in every test in this set to collect data relating to the illuminance, which is the luminous flux incident on a surface measured in lux (lx). This magnitude is related to the distance to the light source and the human perception of light, i.e., only visible wavelengths are measured [43].

Three experiments were proposed with three sunlight scenarios: No Sunlight (NSL), Indirect Sunlight (ISL) and Direct Sunlight (DSL). The season of the year (even month and day), the latitude and weather conditions should be registered, for the sake of rigorous comparison. In this work, the three experiments took place on different sunny days in December 2021, Madrid, Spain.

The NSL experiment consists of a total discharge cycle of the flexible battery, from 100% (battery fully charged) to 0% (battery fully discharged) with no solar cells connected. Some of the performed tests showed that there is no difference between covering the cells so they receive no light and removing them. This experiment serves as a reference to obtain the average current of the system and the battery lifetime without solar energy harvesting.

The ISL experiment follows the same methodology as the NSL experiment (a full discharge cycle) but, in this case, the solar cells were connected to the system and fixed to a window (from the inside), perpendicular to the ground and facing north.

The DSL experiment was similar to the ISL experiment but, this time, the solar cells were placed parallel to the ground so that they could receive sunlight more directly. The solar cells were placed on the eastern side of the building during the morning and on the western side during the afternoon, so that they could receive as much light as possible. Note that direct sunlight is a term that is typically used when incident sun rays are perpendicular to the surface of the solar cell. During this experiment, the solar cells are left under the sun for the whole day, meaning that the incident rays are not perpendicular to their surface the whole time. Even so, in this paper, we will refer to this type of scenario as Direct Sunlight.

### 4.2. Second Set

**Figure 8:**
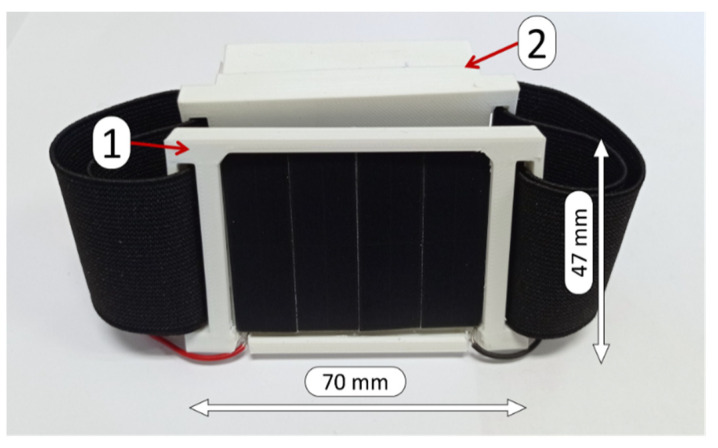
Wearable device prototype. 1: solar cells module housing. 2: PCB and flexible battery housing.

As for the second set of experiments, the designed device was tested as a wearable device by using a two-pieced, 3D-printed, flexible housing, as seen in Figure 8. The prototype is intended to be used as a bracelet, wearing each part of the housing on opposite sides of the wrist or the arm. The first part of the housing (marked as “1” in Figure 8) contains the solar cell semi-flexible module, while the second part of the housing (“2” in Figure 8) contains the PCB and the flexible battery. The user wore the prototype while performing four different Daily Routines (DR) in a city environment. This prototype can be worn easily, as the band is 45 mm in width and parts 1 and 2 are 70 × 47 mm^2^ (a cellular phone is around 70 × 150 mm^2^). Although this device is a prototype and not the final implementation, it has been designed as an accessory, used for street sports, running, jogging, bicycle, etc.

The first Daily Routine (DR1) consisted of wearing the device during two working days with sunny weather in February 2022, Madrid, Spain. The user stayed inside an office during the working day, stayed at home during free time and commuted by public transport. While indoors, the user tried to place the arm wearing the device next to a window when possible. On the other hand, the user tried to walk under the sun as much as possible when outdoors.

The second DR (DR2) consisted of wearing the device during the weekend with cloudy weather in February 2022, Madrid, Spain. The user wore the device at home during free time and outdoors while doing daily errands.

In the third and fourth DR (DR3 and DR4), only two solar cells connected in series were used, so that the dimension of the wearable could be reduced. However, this meant that the power generated by the semi-flexible solar cell module was halved.

In DR3, the device was worn by the user for two working days in April 2022, Madrid, Spain. The first day was sunny and the second day was cloudy. The user wore the device normally while walking outdoors and took the device off to place it under direct sunlight when in the office and stayed still when outside.

In DR4, the device was worn for two working days, both especially sunny, in May 2022, Madrid, Spain. In this case, the user always wore the device and did not purposedly take it off to place it near direct sunlight, but moved the wearing arm next to a window when possible.

### 4.3. Summary

In this subsection, the most important characteristics of every test and other specific details are summarized in Table 1: month and season, the weather during the experiment, the percentage of outdoor time, the number of solar cells used in every test and the position of the solar cell module during the experiment. The first set of experiments were run in 40°16′29.9” N 3°54′26.9” W location. The second set of experiments were run in 40°19′59.5” N 3°45′59.3” W (DR1, DR3 and DR4) and 40°25′0″ N, 3°42′12″ W (DR2).

## 5. Results

In this section, the results of the experiments from the first and second sets of tests are discussed.

### 5.1. First Set

After the three experiments, the data collected from the lux meter (illuminance) and from the battery monitor sent to the mobile phone wirelessly through BLE protocol (current and State-of-charge, SOC) were analyzed.

**Figure 9:**
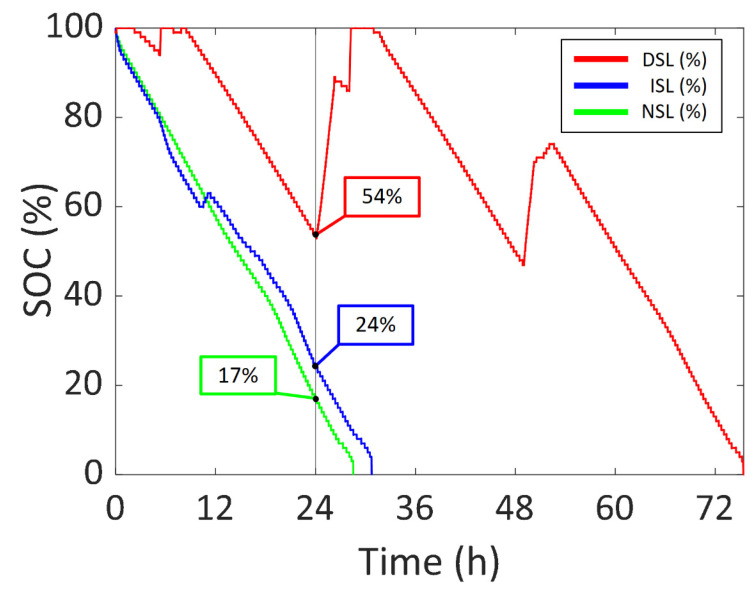
SOC vs. time of the first set of experiments.

In Figure 9, the SOC vs. time of the NSL (No Sunlight), ISL (Indirect Sunlight) and DSL (Direct Sunlight) experiments are presented in green, blue and red, respectively. All curves represent a total discharge of the battery (from 100% to 0%). Thus, the battery lifetime increase can be compared. A vertical line is drawn at 24 h to compare the SOC after a whole day with the different sunlight scenarios. The increases in SOC in every curve are the results of solar cells recharging the battery. Please note that for DSL the exposition to sunlight was terminated after 55 h, leaving the battery to run out. If the experiment had been left working, the energy harvesting circuitry would have continued to recharge the battery. In the NSL scenario, the battery lasted 28.57 h with an average current of −4.2 mA (i.e., the designed device has a power consumption of around 15.5 mW, considering an average battery voltage of 3.7 V). Note that a negative current value means that the battery is discharging. The experiment started at 16:07 and ended at 20:42 the following day. The SOC was 17% 24 h after the start of the experiment.

**Figure 10:**
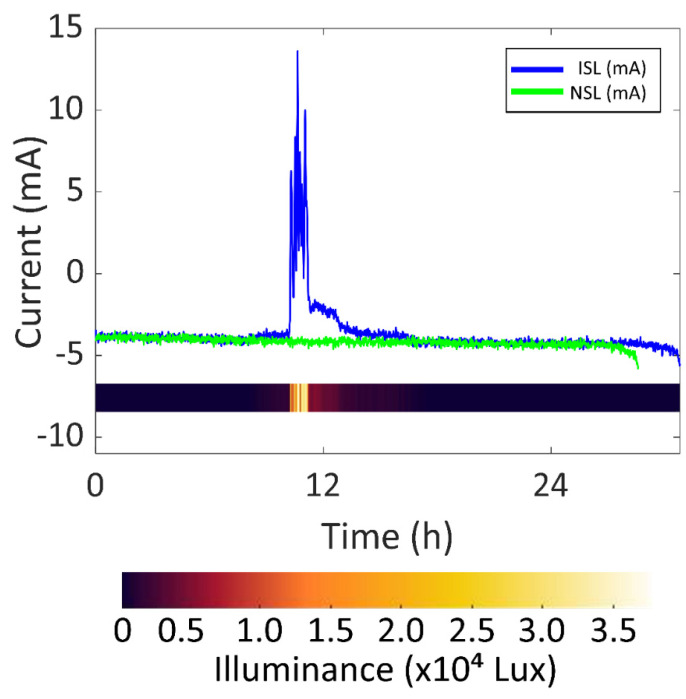
Battery current vs. time of ISL and NSL experiments.

As for the ISL experiment, in Figure 10, the battery current vs. time of both the ISL experiment and the NSL experiment are presented in blue and green, respectively, so that the increase in battery lifetime can be clearly seen. The illuminance vs. time during the ISL experiment is also added to the graph as a colour scale. The battery life reached 30.78 h in the ISL experiment. It started at 00:33 and ended at 7:20 the following day. The solar cells received a significant amount of sunlight for 1.61 h during the experiment, which is 1.25 h per day. It is considered that the solar cells were receiving a significant amount of sunlight whenever the battery current surpasses −2.1 mA (half the average current in the NSL experiment). The solar cells were able to recharge the flexible battery for 0.81 h, i.e., 0.63 h per day. This time was calculated considering that the battery is charging when the measured current is positive. The maximum battery current was 13.6 mA (50.3 mW, considering an average battery voltage of 3.7 V) with an illuminance of 32,000 lux. After 24 h from the start, the SOC was 24%.

**Figure 11:**
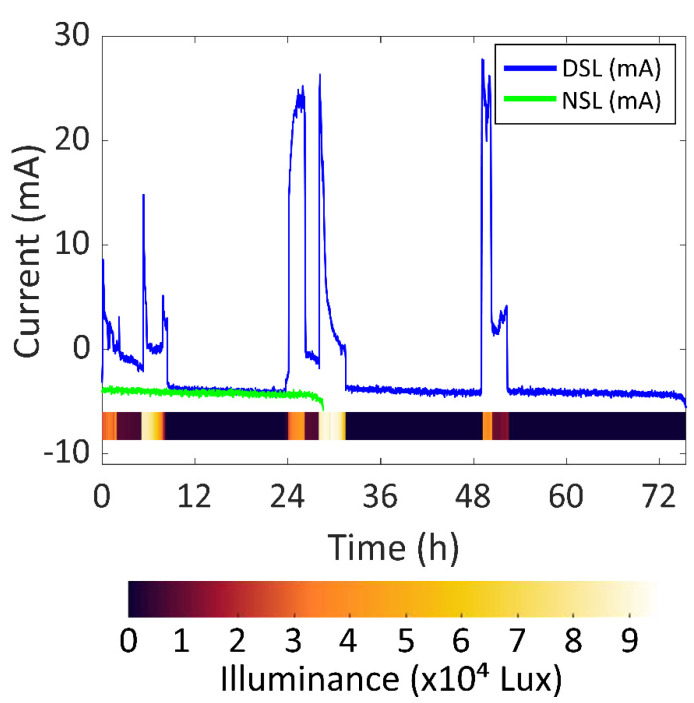
Battery current vs. time of DSL and NSL experiments.

As for the DSL experiment, in Figure 11, the battery current vs. time of the DSL and NSL experiments are presented in blue and green respectively, so that the battery lifetime increase can be compared. Moreover, the illuminance vs. time during the DSL experiment is represented as a colour scale to gain information relating to the amount of light hitting the solar cells module. In this case, the battery lasted 75.42 h. As previously stated, the solar cells were covered after 55 h because the system reached an SOC of 100% in the first two days, as seen in Figure 9. This means that the device could have been permanently working under the same sunlight conditions.

The experiment started at 9:00 and was concluded three days later at 12:43. During the 75.42 h of the experiment, there was a total of 19.00 h of a significant amount of sunlight and the battery was recharged for 13.01 h, i.e., 6.05 h of a significant amount of light and 4.14 h of recharging per day. This means that the system would be able to constantly work if it received significant direct sunlight for around 6 h every day. The highest battery current was 27.8 mA (102.9 mW, considering an average battery voltage of 3.7 V) with 40,870 lux. After 24 h, the SOC was 54%.

### 5.2. Second Set

To examine the results of the three experiments from the second set (Daily Routines, DR), the data obtained from the mobile phone (battery current and SOC sent by BLE from the device) were analyzed.

**Figure 12:**
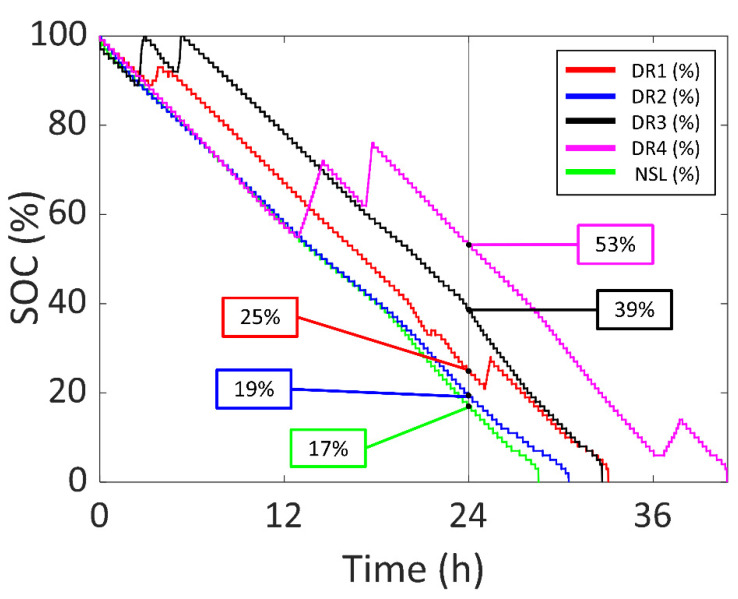
SOC vs. time of the second set experiments and NSL.

In Figure 12, the SOC of the experiments for four Daily Routines (DR1, DR2, DR3 and DR4) are presented in red, blue, black and magenta (respectively), compared to the NSL experiment in green. Furthermore, a vertical line is drawn at 24 h to compare the SOC after a whole day with the different DRs. In DR1, there were increases in the SOC due to using the device outdoors and receiving enough sunlight to recharge the battery. In the DR2 experiment, there was no increase in the SOC because of the cloudy weather. The sunlight outdoors was not able to recharge the battery; however, it was able to decrease current consumption and increase the battery life compared to NSL. In the DR3 experiment, there were two increases in the SOC at the start due to the user purposedly placing the device under the sun while being outdoors and in the office. On the second day, the user repeated the same process, but the device was not able to recharge the battery due to the cloudy weather. In DR4, the first and third increases were due to placing the arm next to a window and the second peak was due to outdoor lighting.

**Figure 13:**
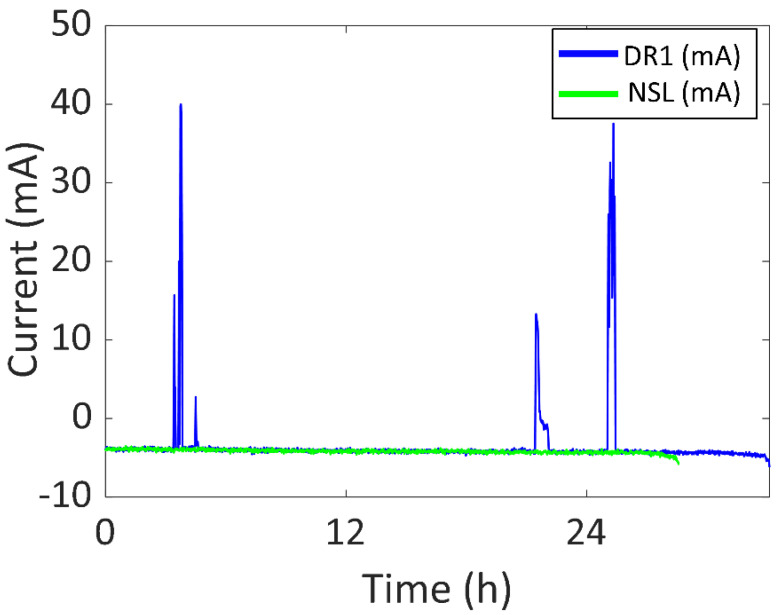
Battery current vs. time of DR1 and NSL experiments.

As for the first Daily Routine (DR1), the battery current vs. time of DR1 compared to the NSL experiment is shown in Figure 13 (DR1 in blue and NSL in green). The experiment started at 12:38 of day one and ended at 21:44 of day two (33.11 h). There was an increase of 4.54 h in the battery life (compared to NSL), with 1.37 h of a significant amount of sunlight (0.99 h per day). The battery was recharged for 0.93 h (0.68 h per day). Three main peaks can be appreciated in the blue curve. The first one was due to the outdoor sunlight received by the device when commuting from work to home. The highest battery current value was reached at this time (40 mA, 148.0 mW). The second peak (13 mA, 48.1 mW) was due to the user placing the wearing arm next to a window while being indoors (office). The third peak (37 mA, 136.9 mW) was due to staying outdoors during a lunch break. After 24 h, the SOC was 25%.

**Figure 14:**
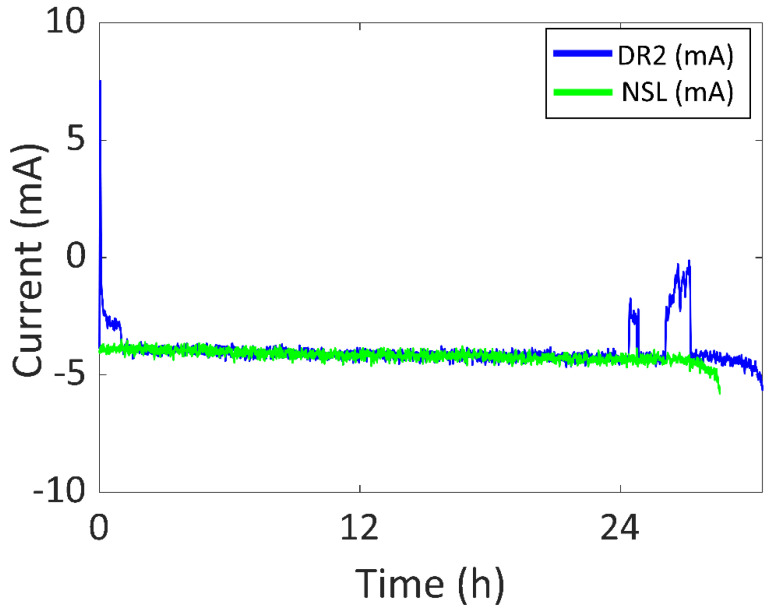
Battery current vs. time of DR2 and NSL experiments.

On the other hand, in Figure 14, the battery current vs. time of both DR2 and NSL experiments are presented in blue and green, respectively. The experiment started at 10:24 of day one and ended at 16:56 of day two (30.54 h). In the DR2 experiment there was an increase of 1.96 h in battery life, compared to NSL. There was a significant amount of sunlight for 1.14 h (0.89 h per day) and the battery was recharged for 0.06 h (0.05 h per day). Note that, even if the light is not enough to charge the battery, decreasing the current consumption also contributes to an increase in the battery life. Three main peaks can be seen in Figure 14. The highest battery current value is 7.5 mA (27.8 mW) due to the user purposedly placing the wearable directly facing the sun. The second and third peaks have a value of −2 mA and 0 mA, respectively. These are due to walking outdoors in a city environment. Note that, compared to DR1, the maximum values of the peaks are lower due to the cloudy weather. After 24 h, the SOC was 19%.

**Figure 15:**
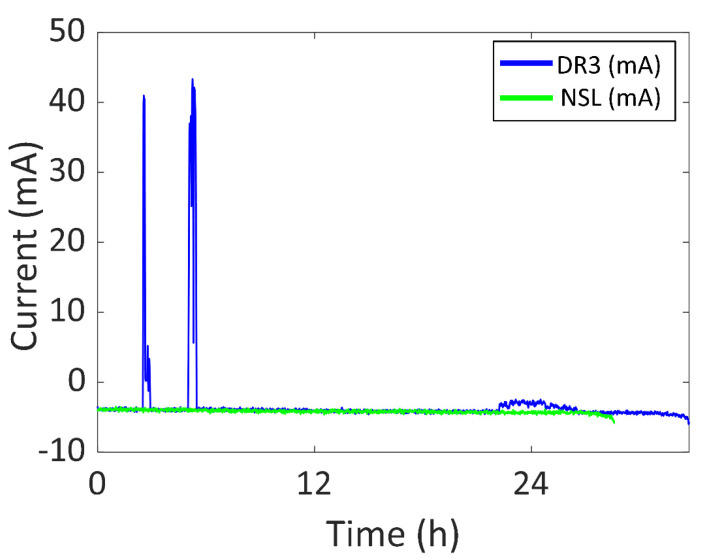
Battery current vs. time of DR3 and NSL experiments.

As for the third Daily Routine (DR3), the battery current vs. time of DR3 compared to the NSL experiment is shown in Figure 15 in blue and green, respectively. The experiment started at 11:30 of day one and ended at 20:13 of day two (32.72 h). There was an increase of 4.14 h in the battery life (compared to NSL), with 0.85 h of a significant amount of sunlight (0.63 h per day). The battery was recharged for 0.79 h (0.58 h per day). The two peaks in the blue curve (41 mA (151.7 mW) and 43 mA (159.1 mW), respectively) are due to the user purposefully taking off the device and placing it under direct sunlight. There is another increase in the battery current 22 h after the start of the experiment (−2.5 mA) due to the user taking the device off again and placing it under the sunlight. However, it was not enough to recharge the battery due to cloudy weather. After 24 h, the SOC was 39%.

**Figure 16:**
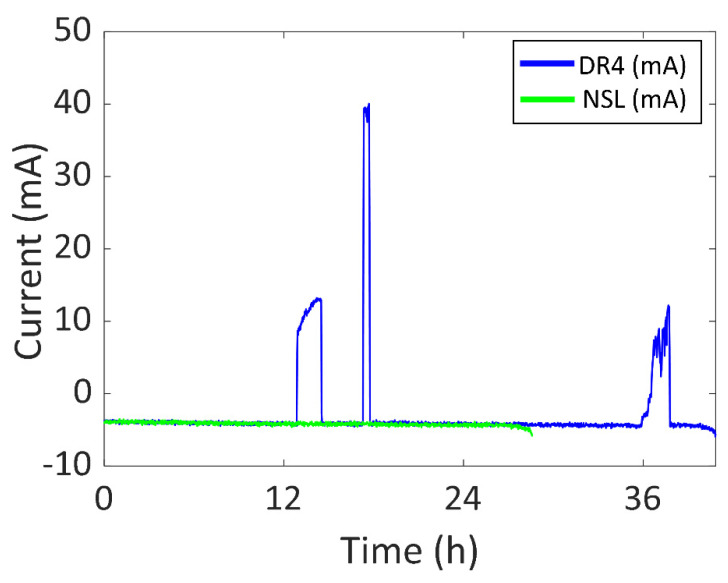
Battery current vs. time of DR4 and NSL experiments.

In Figure 16, the battery current vs. time of DR4 compared to NSL is presented in blue and green respectively. The experiment started at 20:23 of day one and ended at 13:13 of day three, which is a total of 40.84 h, an increase of 12.26 h. There was 3.54 h of a significant amount of sunlight (2.08 h per day) and the battery was recharged for 3.31 h (1.95 h per day). The first and third peaks in the blue curve were due to placing the wearing arm next to a window during a meeting (13 mA (48.1 mW) and 12 mA (44.4 mW), respectively). The second peak was due to staying under outdoor sunlight after lunch (40 mA (148 mW)). After 24 h, the SOC is 53%.

### 5.3. Discussion

In this subsection, the results obtained in the experiments are discussed and compared between them and with other studies in the literature.

In Table 2, the results obtained in this section for all the experiments are presented. These results include the total time in hours of each experiment, the SOC after 24 h, the number of hours with significant amount of sunlight every day (when current surpasses −2.1 mA) and the amount of charging hours due to enough sunlight every day (when current is higher than 0 mA).

These results may seem predictable (battery lifetime increases when adding an energy harvesting system to a wearable). However, the values cannot be easily deduced from the datasheets of the commercial components. It is very difficult to characterize the sunlight conditions of a wearable device due to the high number of variables that must be considered (e.g., user’s movement, weather, solar cells position, country, season, wireless communication connectivity, etc.) For this reason, it was necessary to try to characterize the solar energy harvesting system included in a wearable device by using it in different real-life scenarios, such as the ones presented in this work.

Note that experiments DR3 and DR4 have similar or better results than DR1 and DR2, even when the number of solar cells was halved. DR3 and DR4 were performed in spring, so the incidence angle of sun rays was more favorable. Thus, we can conclude that the performance of the solar energy harvesting system is valid even for winter, and is improved in the other seasons.

Moreover, it is also proven that solar energy is the most efficient technology for a wearable device. The generated power in every test is in the order of the milliwatts, which is higher than most values presented in Section 2 (Related Work). For example, in [28,30], TEGs and piezoelectric devices were used for energy harvesting and achieved results in the order of the microwatts. TEGs need a high temperature difference to generate the required power, which is difficult to accomplish in terms of body temperature and environment temperature. It is possible to increase the generated power by using heat sinks, but they are normally too big to use in a wearable device. TEGs are commonly used in industrial environments, since the machinery and equipment can reach high temperatures compared to the ambient. As for piezoelectric devices, they usually need high frequencies to generate enough power, but human movements normally have low frequencies.

When compared to other solar energy harvesting systems in the literature, the device presented in this work is also proven to have a good performance. In [31], for example, the presented jacket with 16 solar cells and 12 TEGs can generate up to 500 mW (mostly by solar energy), but 16 solar cells are too many for the smart bracelet in Bindi. As for [32], a smart, multi-sensor bracelet, similar to the one in Bindi, is also presented. The device also includes the energy harvesters in its band, including the heat sinks of the TEGs, which could make the bracelet slightly uncomfortable. The average generated power is in the order of the hundreds of microwatts.

Therefore, energy harvesting for wearable devices in IoT systems through other technologies different to solar cells is not competitive. Even in the literature, the approaches proposed with the newest techniques and/or materials are not applicable to wearable devices, which require energies in the range of mW.

## 6. Conclusions

In this work, a device to test the viability of solar-powered wearable devices, including solar energy harvesting in smart wearables with a high number of functionalities, specifically the smart bracelet in Bindi system, has been presented. Furthermore, a procedure for testing the performance of the proposed system has been defined, including a flexible housing 3D-printed design, to easily test the prototype in real situations, since theoretical results are difficult to obtain due to the high number of variables that must be considered (e.g., weather, country, user’s movement, etc.)

In real-life scenarios, the device was able to generate a minimum power of 27.8 mW and a maximum power of 159.1 mW.

The solar energy harvesting system was tested under different light conditions: NSL (No Sunlight), ISL (Indirect Sunlight) and DSL (Direct Sunlight). The SOC after 24 h was 3.18 times higher than NSL in the DSL scenario and 1.41 times higher in the ISL scenario. The maximum generated power was 50.5 mW in ISL and 102.9 mW in DSL. It was concluded that the device could permanently work if the solar cells received a significant amount of DSL for 6 h every day.

Furthermore, the device was also tested in four different Daily Routines (DR) so that the experiment was similar to common use. In DR1, the device was worn during working days in winter with sunny weather and the battery life was 1.16 times higher than NSL. The maximum generated power was 148.0 mW. As for DR2, the device was worn during the weekend in winter with cloudy weather and the battery life was 1.07 times higher than NSL. The maximum generated power was 27.8 mW. To reduce the dimensions of the prototype, two alternative DRs were included in this set (DR3 and DR4) using only two solar cells in the semi-flexible module. In DR3, the device was used during both a sunny and a cloudy working day in spring and the battery life was 1.14 higher than NSL. The maximum generated power was 159.1 mW. In DR4, the device was used for three working days in spring and the battery life was 1.43 times higher than NSL. The maximum generated power was 148.0 mW.

It can be concluded that, even when the number of solar cells is halved, the season has a very high influence on the results.

The dimensions of the presented proof-of-concept study are not optimal for a wearable device. However, it was proven that there is an important contribution to battery life even if only two solar cells are used. The second part of the prototype (PCB and flexible battery) can be easily reduced, since the PCB layout can be optimized and the flexible battery can adapt its shape around the wrist.

The users of the device know about the possibility of recharging the battery with solar energy, so they can deliberately place the device under a significant amount of light to charge it (e.g., sitting next to a window when indoors or changing the device position so that it receives more direct sunlight when outdoors).

On the other hand, commercial devices have been used in this design. Even if there has been a rapid increase in energy harvester development, most of them are experimental and they are usually hard to produce on a large scale because of the fabrication processes and costs.

The main conclusion of this work is that using solar energy harvesting with commercial solar cells and a commercial flexible LiPo battery as an ESD is a viable solution to increase battery life in wearables with intense data processing and wireless communications, although a rigorous characterization should be done depending on the latitude, weather, wireless connectivity and users’ daily routines.

## Figures and Tables

**Table 1:** Summary of the characteristics of the tests.

Set	Test	Month (Season)	Weather	% Outdoor Time	Number of Solar Cells	Position of Solar Cells
1	NSL	December (Winter)	-	-	-	-
	ISL	December (Winter)	Sunny	-	4	Window
	DSL	December (Winter)	Sunny	-	4	Window
2	DR1	February (Winter)	Sunny	9	4	Wrist
	DR2	February (Winter)	Cloudy	25	4	Wrist
	DR3	April (Spring)	Sunny/cloudy	12	2	Wrist *
	DR4	May (Spring)	Sunny	12	2	Wrist

* The user purposedly took the device off and place it under the sun when possible.

**Table 2:** Time results for all experiments.

	NSL	ISL	DSL	DR1	DR2	DR3	DR4
Total time (h)	28.58	30.78	75.42	33.11	30.54	32.72	40.84
SOC after a day (%)	17	24	54	25	19	38	53
Sunlight time per day (h/day)	-	1.25	6.05	0.99	0.89	0.63	3.54
Charging time per day (h/day)	-	0.63	4.14	0.68	0.05	0.58	3.31
Maximum power (mW)	-	50.3	102.9	148.0	27.8	159.1	148.0

## Data Availability

Not applicable.
